# Assessing the validity of driver gene identification tools for targeted genome sequencing data

**DOI:** 10.1093/bioadv/vbae073

**Published:** 2024-05-23

**Authors:** Felipe Rojas-Rodriguez, Marjanka K Schmidt, Sander Canisius

**Affiliations:** Division of Molecular Pathology, The Netherlands Cancer Institute—Antoni van Leeuwenhoek Hospital, 1066 CX Amsterdam, The Netherlands; Division of Molecular Pathology, The Netherlands Cancer Institute—Antoni van Leeuwenhoek Hospital, 1066 CX Amsterdam, The Netherlands; Department of Clinical Genetics, Leiden University Medical Center, 2333 ZC Leiden, The Netherlands; Division of Psychosocial Research and Epidemiology, The Netherlands Cancer Institute—Antoni van Leeuwenhoek Hospital, 1066 CX Amsterdam, The Netherlands; Division of Molecular Pathology, The Netherlands Cancer Institute—Antoni van Leeuwenhoek Hospital, 1066 CX Amsterdam, The Netherlands; Division of Molecular Carcinogenesis, The Netherlands Cancer Institute—Antoni van Leeuwenhoek Hospital, 1066 CX Amsterdam, The Netherlands

## Abstract

**Motivation:**

Most cancer driver gene identification tools have been developed for whole-exome sequencing data. Targeted sequencing is a popular alternative to whole-exome sequencing for large cancer studies due to its greater depth at a lower cost per tumor. Unlike whole-exome sequencing, targeted sequencing only enables mutation calling for a selected subset of genes. Whether existing driver gene identification tools remain valid in that context has not previously been studied.

**Results:**

We evaluated the validity of seven popular driver gene identification tools when applied to targeted sequencing data. Based on whole-exome data of 14 different cancer types from TCGA, we constructed matching targeted datasets by keeping only the mutations overlapping with the pan-cancer MSK-IMPACT panel and, in the case of breast cancer, also the breast-cancer-specific B-CAST panel. We then compared the driver gene predictions obtained on whole-exome and targeted mutation data for each of the seven tools. Differences in how the tools model background mutation rates were the most important determinant of their validity on targeted sequencing data. Based on our results, we recommend OncodriveFML, OncodriveCLUSTL, 20/20+, dNdSCv, and ActiveDriver for driver gene identification in targeted sequencing data, whereas MutSigCV and DriverML are best avoided in that context.

**Availability and implementation:**

Code for the analyses is available at https://github.com/SchmidtGroupNKI/TGSdrivergene_validity.

## 1 Introduction

Identification of cancer genes from sequencing data is commonly performed to characterize the molecular landmarks of tumor progression (Zhu *et al.* 2020). Mutations in cancer driver genes may trigger tumor growth and spread (Tokheim *et al.* 2016, Zhao *et al.* 2019), and are considered to undergo positive selection during clonal expansion. Although many passenger mutations also undergo clonal expansion, they do not confer strong selective tumor advantages (Dietlein *et al.* 2020). Accordingly, Darwinian principles of variation and selection can be exploited to identify cancer-driver genes (Muinos *et al.* 2021). The main objective of driver gene identification tools is to identify signals of positive selection among the abundance of passenger mutations (Tokheim *et al.* 2016).

Many approaches to driver gene identification were originally developed and tested in the context of whole-exome sequencing (Zhu *et al.* 2020). The Cancer Genome Atlas (TCGA) (The Cancer Genome Atlas Research Network *et al.* 2013) and the International Cancer Genome Consortium (The International Cancer Genome Consortium 2010) produced large amounts of whole-exome or whole-genome sequencing data across multiple cancer types, and were the driving force behind the development of several driver detection tools. However, in recent years, many large sequencing projects have used targeted panel sequencing (Cheng *et al.* 2015, Pereira *et al.* 2016, Hartmaier *et al.* 2017, Pugh *et al.* 2022). The reduced cost of panel sequencing enables the profiling of larger numbers of tumors at greater depth, which increases the potential to detect new signals of positive selection. Targeted sequencing datasets, therefore, create promising new opportunities for driver gene identification.

Since targeted sequencing data differ from whole-exome data in key aspects, it is important to assess whether existing driver gene identification tools can be used outside of their original application domain. In contrast to whole-exome sequencing, targeted sequencing only covers a small fraction of the genome. Therefore, mutations are only identified in a selected number of genes, typically a few hundred, resulting in datasets with far fewer mutations. Since the gene panels are often composed of genes postulated to play a role in cancer, these mutations will include an overrepresentation of driver mutations compared to whole-exome mutation data. Because of these differences, tools developed with whole-exome data in mind need to be validated on targeted sequencing data before they can be used in that context.

Tools for driver gene detection utilize a variety of statistical approaches to identify signals of positive selection (Porta-Pardo *et al.* 2017). An important aspect of many tools is the formulation of a background model that describes mutations not under positive selection. Genomic features, such as a gene’s length, its local epigenetic context, or replication time have been found to correlate with the presence of mutations in genes, even in the absence of positive selection. Therefore, positive selection should only be suspected if the observed accumulation of mutations in a gene exceeds the expectation based on such simpler features. Published tools differ in the types of genomic features used, as well as in their statistical approaches to estimate the effect of those features on background mutation rates. We hypothesized that the choices made for these background models affect the validity of a tool when applied to targeted sequencing data.

Numerous tools leveraging multiple genomic features and statistical models exist to identify driver genes. We investigated seven widely used tools (20/20+ [Tokheim *et al.* 2016], ActiveDriver [Reimand and Bader 2013], dNdScv [Martincorena *et al.* 2017], DriverML [Han *et al.* 2019], MutSigCV [Lawrence *et al.* 2013], OncodriveCLUSTL [Arnedo-Pac *et al.* 2019], and OncodriveFML [Mularoni *et al.* 2016]) as a representation of the various approaches to driver gene detection. Detailed information about the statistical background for each of these tools is provided in Supplementary Table S1. Our main objective was to assess whether these tools produce reliable driver gene predictions when applied to targeted sequencing data. The central assumption in our analyses was that a valid tool for targeted data must produce similar driver predictions regardless of whether whole-exome sequencing or targeted sequencing was used. For this comparison, we used whole-exome mutation data and constructed matching targeted data by intersecting the mutations with the target regions of the MSK-IMPACT gene panel. While agreement between driver gene predictions in both settings was our primary evaluation criterion, we also tested whether the tools failed to identify some known cancer genes as driver genes in analyses of targeted sequencing data.

We studied mutation data for 14 different cancer types to ensure that our conclusions apply to a wide range of biological contexts and mutation profiles. In particular, we were interested whether the validity of the driver gene detection tools on targeted sequencing data depended on the differences in overall mutation load across the cancer types. Furthermore, we investigated whether our conclusions depend on the composition of the gene panel used in targeted sequencing. To this aim, in addition to the pan-cancer MSK-IMPACT panel used for all cancer types, we repeated our analyses of the breast cancer mutation data using the breast cancer-specific B-CAST panel. Here, we present a systematic assessment to test the validity of driver identification tools in the context of targeted genome sequencing.

## 2 Methods

### 2.1 Sample acquisition

We obtained mutation data for cancer types with more than 400 patients available in TCGA. These cancer types were bladder cancer (BLCA; bladder urothelial carcinoma), breast cancer (BRCA; breast invasive carcinoma), colon cancer (COAD; colon adenocarcinoma), glioblastoma (GBM; glioblastoma multiforme), head and neck cancer (HNSC; head and neck squamous cell carcinoma), kidney clear cell cancer (KIRC; kidney renal clear cell carcinoma), lung cancer (LC; lung adenocarcinoma and lung squamous cell carcinoma combined), glioma (LGG; brain lower grade glioma), ovarian cancer (OV; ovarian serous cystadenocarcinoma), prostate cancer (PRAD; prostate adenocarcinoma), melanoma (SKCM; skin cutaneous melanoma), stomach cancer (STAD; stomach adenocarcinoma), thyroid cancer (THCA; thyroid carcinoma), and endometrial cancer (UCEC; uterine corpus endometrial carcinoma). These 14 cancer types had large sample sizes providing sufficient power for our analyses, and covered the mutation load spectrum across cancer types in TCGA (Supplementary Fig. S1).

Whole-exome mutation data for each cancer type were extracted from the TCGA Pan-Cancer Atlas *MC3 MUTATION* file (v0.2.8) and cancer type labels were extracted from the *CLINICAL WITH FOLLOW-UP* file (both files are available from https://gdc.cancer.gov/about-data/publications/pancanatlas). After matching the clinical data and the mutation data, we obtained a total of 369 tumor samples for KIRC, 388 for GBM, 404 for COAD, 411 for BLCA and OV, 438 for STAD, 466 for SKCM, 491 for THCA, 494 for PRAD, 507 for HNSC, 510 for LGG, 530 for UCEC, 1000 for LC and 1020 for BRCA.

### 2.2 Construction of targeted sequencing mutation data

**Figure 1.**
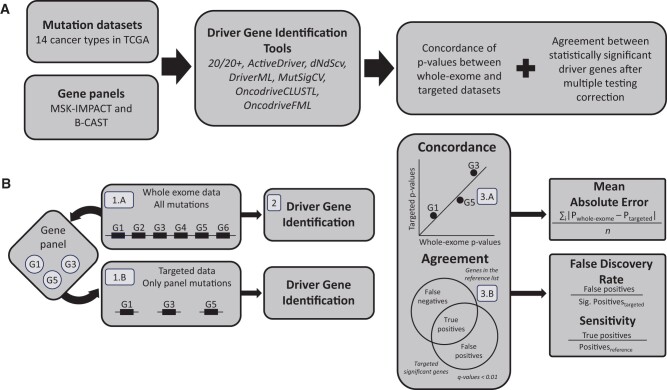
Illustration of the workflow for assessing the validity of driver detection tools on targeted genome sequencing data. (A) Overview of the workflow. Whole-exome sequencing datasets for each of the 14 cancer types were obtained from TCGA. Both B-CAST and MSK-IMPACT gene panels were used to construct matching targeted datasets. Seven driver gene identification tools were applied to both whole-exome and matching targeted datasets. Concordance and agreement of predictions were used to estimate the validity of each tool individually. (B) Details of the workflow for each individual cancer type and gene panel. For each whole-exome dataset (1.A) a matching targeted dataset was constructed (1.B) only containing mutations in the genes composing the gene panel. (2) Each driver gene identification tool was used on each dataset individually. (3.A) Concordance between *P*-values was quantified for each tool using the mean absolute error between whole-exome and targeted datasets. (3.B) Agreement between whole-exome and targeted driver gene predictions was quantified using false discovery rate and sensitivity, using whole-exome as reference, for genes statistically significant after multiple testing correction (Benjamini–Hochberg < 0.01).

We constructed targeted sequencing datasets based on the whole-exome mutation data for all 14 cancer types (Fig. 1). Our primary analyses used the MSK-IMPACT gene panel, but to ensure that our results did not depend on a particular gene panel, we also tested the B-CAST gene panel when analyzing the breast cancer dataset. The MSK-IMPACT panel, which includes 505 genes, was originally designed for pan-cancer data aiming to identify genes involved in multiple cancer types (Cheng *et al.* 2015). In contrast, the B-CAST panel, which includes 323 genes, was specifically designed for breast cancer (B-CAST Gene Panel Development Team 2023).

For a given gene panel, we constructed matching targeted mutation datasets by only including mutations from the whole-exome dataset that overlap with the panel genes based on gene symbols. To do so, we used the assignment of mutations to genes as provided by TCGA. Individual mutations were retained in the targeted dataset if the assigned HUGO gene symbol matches one of the genes in the panel. All other mutations were removed. For all cancer types, targeted datasets were constructed using the MSK-IMPACT gene panel. In addition, for breast cancer, we also created a targeted dataset based on the B-CAST gene panel.

### 2.3 Driver gene identification tools

A total of seven tools were used. We included an overview of the methodological details of each tool focusing on the computation of the gene scores and their null distribution in Supplementary Table S1. All analyses used hg19 as the reference genome.

- **20/20+**: 20/20+ (v1.2.3) (Tokheim *et al.* 2016). 20/20+ was run using the Snakemake workflow provided by the 20/20+ authors. We used the 100k training classifier for all samples. 20/20+ uses the pre-computed scores based on reference transcript annotation from SNVBox (Weckx *et al.* 2004).
- **ActiveDriver**: ActiveDriver (R-based: v1.0.0) (Reimand and Bader 2013). We used the default parameters for both whole-exome and targeted samples. ActiveDriver uses phosphorylation data from PhosphoSitePlus (Hornbeck *et al.* 2012), PhosphoELM (Dinkel *et al.* 2011), and Human Protein Reference Database (Keshava Prasad *et al.* 2009). We excluded all genes with *unavailable sequence* label in the fasta sequences (ens70_protein_seqs.fa.rsav file) and the corresponding entry for disorder sequences (ens70_protein_seqs_disorder.fa.rsav file).
- **dNdScv**: dNdScv (R-based: v0.0.1.0) (Martincorena *et al.* 2017). We analyzed whole-exome data using default settings. For targeted data, we specified a list of HUGO symbols for the corresponding panel. We did not set limits for the maximum number of mutations per gene for each sample and the maximum number of coding mutations per sample.
- **DriverML**: DriverML (v1.0) (Han *et al.* 2019). We used the default parameters for both whole-exome and targeted datasets.
- **MutSigCV**: MutSigCV (v1.41) (Lawrence *et al.* 2013). We used the coverage (exome_completeTCGA_full192.coverage), dictionary (mutation_type_dictionary_file), and covariate (gene.covariates) files included in MutSigCV by default.
- **OncodriveCLUSTL**: OncodriveCLUSTL (v1.1.3) (Arnedo-Pac *et al.* 2019). We set the simulation mode parameter to *region-restricted* to normalize mutation frequency in the regions provided. We set the explicit concatenation parameter to calculate clustering on neighboring exons for both whole-exome and targeted samples. We set the computation of mutation probabilities (signature calculation) to *region-normalized* to only consider reference counts in the regions available. We used the default number of simulations and only considered the analytical *P*-values in our analyses.
- **OncodriveFML**: OncodriveFML (v2.2) (Mularoni *et al.* 2016). We configured the sequencing parameter to distinguish between the targeted and whole-exome sequencing data in the application of the tool. For whole-exome, we set the parameter to *wes*. For targeted data, we set the sequencing parameter to *targeted*, meaning that no normalization by site is performed. We used the genomics scores of CADDpack version 1.0. We set the method of mutation signature to *complement* for both whole-exome and targeted, meaning that the matrix of 96 possible changes of trinucleotides was used. We set the sampling number to $1\times 10^{6}$ with a maximum of $1\times 10^{7}$, sampling chunk to 200, and sampling minimal observations to 20.

### 2.4 *P*-value concordance between whole-exome and targeted sequencing predictions

To assess the validity of a driver detection tool for targeted sequencing data, we compared the panel genes’ *P*-values estimated from the whole-exome data with the *P*-values from the matching targeted sequencing datasets. In doing so, our assumption was that valid tools estimate similar *P*-values for both targeted datasets and whole-exome data. We performed comparisons between the targeted sequencing dataset and the matching whole-exome dataset for each individual cancer type.

For a high-level inspection of a tool’s *P*-value concordance, we visually compared the panel genes’ *P*-values using scatter plots. In addition, we quantified the concordance of *P*-values by computing the mean absolute error (MAE) across all gene-matched *P*-values, defined as: $\text{MAE}=\sum_{i}\left|p_{i}^{\text{WE}}-p_{i}^{\text{TS}}\right|/n$, where $p_{i}^{\text{WE}}$ and $p_{i}^{\text{TS}}$ are the estimated *P*-values for gene *i* based on whole-exome and targeted sequencing data respectively, and *n* is the total number of panel genes tested. A high *P*-value concordance corresponds to a low (ideally 0) MAE.

### 2.5 Agreement on detected driver genes between whole-exome and targeted sequencing

Although we generally expect that tools valid for targeted sequencing data will show a high *P*-value concordance, such concordance is not strictly required across the entire range of *P*-values. For practical purposes, it only matters that the whole-exome and targeted sequencing analyses agree on the set of statistically significant driver genes. We therefore performed a complementary analysis to quantify this agreement. Panel genes were classified as significant driver genes, separately for both the whole exome and targeted sequencing data. Significance was determined after correction for multiple testing using a Benjamini–Hochberg false discovery rate threshold of 0.01. Importantly, the multiple testing correction for whole-exome *P*-values was applied across the panel genes only. Next, taking the whole-exome driver genes as the reference, we quantified the agreement on statistically significant driver genes using the following two metrics:

$$\begin{array}{c} \text{False discovery rate}=\text{FP}/P^{\text{TS}}\\ \text{Sensitivity}=\text{TP}/P^{\text{REFERENCE}} \end{array}$$

where

FP = false positives, i.e. the number of significant driver genes based on the targeted sequencing data that are not significant based on the whole-exome data

TP = true positives, i.e. the number of significant driver genes based on both the whole-exome and the targeted sequencing data

$P^{\text{TS}}$ = targeted sequencing positives, i.e. the number of significant driver genes based on the targeted sequencing data

$P^{\text{RERERENCE}}$ = whole-exome positives, i.e. the number of significant driver genes based on the whole-exome data

A high concordance corresponds to a low (ideally 0) false discovery rate and high (ideally 1) sensitivity. For both the false discovery rate and the sensitivity metrics, we computed a 95% confidence interval using the method proposed by Clopper and Pearson (1934).

### 2.6 Known cancer driver genes

To evaluate predicted driver genes against known cancer driver genes, we constructed cancer-type-specific driver gene sets. We extracted lists of driver genes specific for each of the 14 cancer types from the cancer gene census (CGC). Selected genes had to meet the following three conditions. (i) The gene was marked as Tier 1, indicating there is well-documented evidence of its role in cancer along with evidence supporting that its mutations are associated with oncogenesis. (ii) The mutation type linked to the gene’s driver role was either frameshift, missense, nonsense, or splice site mutation. This condition excluded genes primarily altered by broader copy number changes, which cannot be detected by the tools we evaluated. (iii) There is evidence for the gene’s involvement in the particular cancer type. For this, we matched the tumor-type annotation in CGC to the 14 cancer types based on Supplementary Table S2.

## 3 Results

### 3.1 Validity of driver gene identification tools for targeted genome sequencing datasets

**Figure 2.**
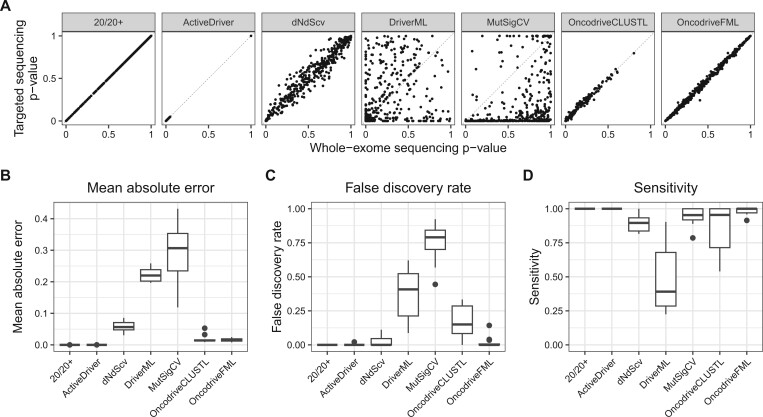
Comparison of driver identification results based on whole-exome and targeted datasets. The tools used were 20/20+, ActiveDriver, dNdScv, DriverML, MutSigCV, OncodriveCLUSTL, and OncodriveFML. (A) *P*-value concordance visualized by scatter plots comparing gene *P*-values obtained using whole-exome sequencing data to those obtained using the matched targeted sequencing data. The dashed diagonal line indicates optimal concordance. The results shown here are for the breast cancer mutation data. Full results for all 14 cancer types are shown in Supplementary Figure S2. (B) *P*-value concordance quantified by the mean absolute errors of *P*-values, summarized by driver detection tool. Each box describes the results across all 14 cancer types. Detailed statistics per cancer type are shown in Supplementary Table S3. (C) Agreement in predicted driver genes, evaluated using false discovery rate, summarized by driver identification tool. Each box describes the results across all 14 cancer types. Detailed statistics per cancer type are shown in Supplementary Table S4. (D) Agreement in predicted driver genes, evaluated using sensitivity, summarized by driver detection tool. Each box describes the results across all 14 cancer types. Detailed statistics per cancer type are shown in Supplementary Table S4.

We applied the seven driver gene identification tools to the whole-exome mutation data of the 14 cancer types and to the constructed matching targeted datasets individually. For each tool and for each dataset, concordance of *P*-values between whole-exome and targeted data was used to assess whether the two types of sequencing data showed consistent estimates (Fig. 2A and B, Supplementary Fig. S2 and Table S3). These comparisons revealed clear differences in the validity of each tool on targeted sequencing data.

ActiveDriver and 20/20+ displayed near-perfect *P*-value concordance, with error values close to 0 (Supplementary Table S3). We observed that the *P*-values for ActiveDriver clustered around either 0 or 1, with no *P*-values in between. This distribution was different from any of the other tools and seems at odds with the assumption that *P*-values are distributed uniformly under the null hypothesis. However, similar results for ActiveDriver have been observed in other studies (Tokheim *et al.* 2016). Moreover, we observed this particular clustering of *P*-values for both the whole-exome and targeted sequencing data. With the negligible errors of both ActiveDriver and 20/20+, driver predictions on targeted sequencing data match those on whole-exome data.

For OncodriveFML (MAE ranging from 0.008 to 0.025), OncodriveCLUSTL (MAE from 0.009 to 0.05), and dNdScv (MAE from 0.03 to 0.086), the concordance between whole-exome and targeted data was not perfect, yet we still observed an adequate p-value concordance. Especially for OncodriveFML and OncodriveCLUSTL, the error was relatively small. The results for dNdScv showed larger errors, but generally, there was a clear correlation between the *P*-values for whole-exome and targeted data (Fig. 2B). Notwithstanding these small errors, complementary analyses were required to assess the extent to which driver predictions on targeted data will match those on whole-exome data.

MutSigCV (MAE from 0.12 to 0.43) and DriverML (MAE from 0.20 to 0.26) had a high discordance in *P*-values across all datasets. Notably, the targeted sequencing *P*-values for MutSigCV were extremely skewed towards 0 while the matching whole-exome *P*-values ranged between 0 and 1. DriverML seemed to adequately estimate the predictions for a subset of genes resulting in predictions similar between targeted and whole-exome sequencing datasets. However, the errors observed across all genes showed an overall poor concordance, with both severe under-estimation and over-estimation of *P*-values (Supplementary Fig. S2).

### 3.2 Agreement on detected driver genes in targeted datasets

In addition to the concordance of *P*-values, we assessed the agreement on statistically significant driver genes between the whole-exome and targeted sequencing analyses (Fig. 2C and D; Supplementary Table S4). As expected, the near-perfect *P*-value concordance of ActiveDriver and 20/20+ was reflected by a near-perfect false discovery rate and sensitivity. Likewise, the three tools that had reasonably good *P*-value concordances (i.e. dNdScv, OncodriveFML, and OncodriveCLUSTL) also showed reasonably good agreement on driver predictions, although we observed differences with respect to their false discovery rate and sensitivity. OncodriveFML driver predictions mostly matched those on whole-exome data, but with a few false positive genes increasing the false discovery rate and a few false negative genes decreasing sensitivity. OncodriveCLUSTL had a wider range of values for some cancer types. We observed that the majority of cancer types displayed excellent sensitivity and specificity, yet we saw low sensitivity for BRCA, HNSC, LC, LGG, and STAD, and high false discovery rate for BRCA, COAD, GBM, PRAD, and SKCM (Supplementary Table S4). However, by using a less restrictive critical value for multiple testing (Benjamini–Hochberg < 0.05) both false discovery rate and sensitivity improved for the previously mentioned cancer types, except for GBM and COAD where the false discovery rate remained largely unchanged (Supplementary Table S5). dNdScv achieved a near-perfect false discovery rate, despite the relatively large errors in the *P*-value concordance analysis. However, the latter did cause slightly lower sensitivity across cancer types (Fig. 2D).

We observed that the two tools with poor concordance also showed low agreement on statistically significant driver predictions. MutSigCV had an almost optimal sensitivity (close to 1 across all cancer types), but this came at the price of extremely high false discovery rates (ranging from 0.44 to 0.92). This conforms with the observation that MutSigCV’s *P*-values were heavily skewed towards 0 on the targeted sequencing data. DriverML generally showed both a high false discovery rate (ranging from 0.09 to 0.62) and a low sensitivity (ranging from 0.22 to 0.90) across cancer types.

### 3.3 Effect of mutation load

**Figure 3.**
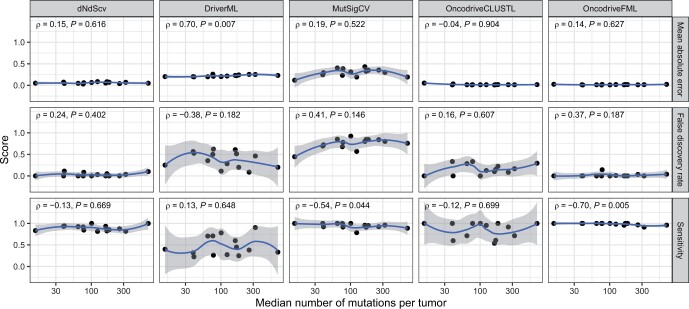
Effect of tumor mutation load on *P*-value concordance and driver gene agreement. Dots represent the 14 cancer types. The *x*-axes are on a logarithmic scale and correspond to a cancer type’s median number of mutations per tumor. The *y*-axes correspond to the mean absolute error (for *P*-value concordance), and the false discovery rate and sensitivity (for driver gene agreement). The blue lines and gray-shaded areas were obtained using a loess fit, and are strictly shown for visual guidance. Correlation coefficients ρ and their *P*-values were estimated using Spearman correlation. 20/20+ and ActiveDriver have been omitted from the plot since they showed near-perfect concordance and agreement for all cancer types.

The 14 cancer types in our study represent a wide range in tumor mutation loads. We were interested in whether these differences in average mutation load affect how well a tool performs on targeted sequencing data. Therefore, we correlated the mean absolute error, false discovery rate, and sensitivity metrics across cancer types to the median tumor mutation load of those cancer types. An increasing or decreasing trend for one of the metrics would suggest that tumor mutation load affects a tool’s potential on targeted sequencing data. However, we did not observe a strong trend for any of the tools and any of the metrics (Fig. 3). While DriverML’s mean absolute error and OncodriveFML’s sensitivity showed a significant correlation with tumor mutation load, the magnitude of change over the entire range of mutation loads was too small to be of practical relevance. No other tool showed a dependence on tumor mutation load.

### 3.4 Features correlated with discordant driver gene predictions

**Figure 4.**
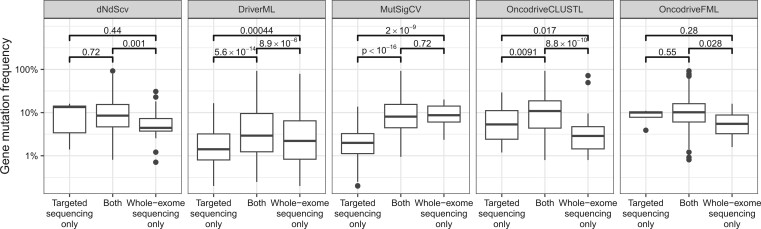
Comparison of the mutation frequencies of genes identified exclusively in the targeted sequencing or whole-exome datasets, or in both. Results for all 14 cancer types were aggregated. *P*-values were obtained with the Wilcoxon rank sum test. ActiveDriver and 20/20+ were not included because the exact same sets of genes were identified in the targeted sequencing dataset and in the whole-exome dataset.

We investigated whether disagreement on detected driver genes in the whole-exome and targeted analyses could be explained by specific features of the genes that are classified differently in the two analyses. Specifically, we considered the effect of gene length and gene mutation frequency within the dataset. We observed no differences in gene length between genes that were uniquely identified in either the targeted sequencing or the whole-exome sequencing datasets (Supplementary Fig. S3). In contrast, differences in gene mutation frequency did correlate with discordant predictions for some of the tools (Fig. 4). For DriverML and MutSigCV, genes only classified as drivers in the targeted datasets had a significantly lower mutation frequency than genes only detected in the whole-exome datasets or genes detected in both datasets. A smaller difference was observed for OncodriveCLUSTL, where we found that genes that were uniquely identified in the targeted sequencing dataset had, on average, a higher mutation frequency than genes only identified in the whole-exome dataset.

### 3.5 Effect of gene panel composition

We repeated the breast cancer analyses using the breast cancer-specific B-CAST panel instead of the pan-cancer MSK-IMPACT panel. Doing so, we could assess whether our previous observations would change if we used a gene panel specifically designed for the cancer type studied. The general concordance and agreement metrics for the seven tools remained fairly consistent (Supplementary Tables S3 and S4). The three tools with high false discovery rates using the MSK-IMPACT panel, DriverML, MutSigCV, and OncodriveCLUSTL, still had high false discovery rates using the B-CAST panel. DriverML and dNdScv had reduced sensitivity using the MSK-IMPACT panel, which remained unchanged using the B-CAST panel. OncodriveCLUSTL had reduced sensitivity in the MSK-IMPACT analysis, whereas it had perfect sensitivity with the B-CAST panel. The confidence intervals for both panels overlapped though.

Next, we looked at the overlap of detected driver genes using the two gene panels. In doing so, we accounted for the fact that only genes that are in both panels could have been detected in both analyses. All seven tools largely detected the same sets of driver genes (Supplementary Table S6), regardless of which panel was used for the targeted sequencing. The largest differences were seen for DriverML and MutSigCV, although these differences are still small relative to the large numbers of genes they detected.

### 3.6 Comparison to known cancer driver genes

As a complementary analysis, we tested whether some known cancer driver genes were only detected in either the whole-exome or targeted sequencing data. For each tool and each cancer type, we composed two sets of genes: those only detected as driver in the whole-exome data, and those only detected as driver in the targeted data. For both gene sets, we counted the overlap with a list of known driver genes for that particular cancer type based on the Cancer Gene Census (CGC). Importantly, the MSK-IMPACT panel is composed of genes suspected or known to be important in cancer. If the set of driver genes detected in one setting but not in the other is large enough, there is likely to be some overlap with the CGC purely by chance. We therefore tested the overlap with CGC for enrichment using a Fisher’s exact test.

Since ActiveDriver and 20/20+ had perfect concordance, they showed no differences in detected known drivers. For all other tools, we observed that some known drivers were only detected in the whole-exome data, whereas other drivers were only detected in the targeted data (Supplementary Tables S7 and S8). Focusing on the cases where the Fisher’s exact test indicated a significant enrichment, differences mostly involved only one or two genes. DriverML is the exception, identifying five know driver genes uniquely in the targeted data for lung cancer, and eight known driver genes uniquely in the whole-exome data for colon cancer. The more general question of whether the drivers predicted by the various tools were in fact bona fide cancer driver genes was outside of the scope of this study. This question has been addressed elsewhere (Porta-Pardo *et al.* 2017, Bailey *et al.* 2018, Martinez-Jimenez *et al.* 2020).

## 4 Discussion

Effective identification of driver genes relies on rigorous application of driver detection methods. We tested seven driver identification tools in the context of mutation data obtained by targeted sequencing. Our goal was to evaluate how well their identified driver genes matched the genes identified in whole-exome data, the setting for which the tools were originally developed. The results showed that only two of the tools, 20/20+ and ActiveDriver, had perfect concordance on targeted sequencing data. Three other tools, OncodriveFML, OncodriveCLUSTL, and dNdScv, reached an acceptable level of concordance. In contrast, two tools, MutSigCV and DriverML produced results that were highly discordant with their whole-exome results. These observations were consistent across 14 different cancer types, and in the case of breast cancer, for two different gene panels.

To explain why certain tools perform well on targeted sequencing data while others do not, it is informative to look at their underlying statistical models. Most tools assess whether a gene carries sufficient evidence of positive selection by comparing the observed mutations to a null distribution based on the assumption that all of them are passenger mutations. Among the tools we tested, we distinguish two general approaches to estimating that null distribution: within-gene approaches, which estimate the distribution using only the observed mutations for each individual gene, and across-genes approaches, which estimate the distribution using mutations across multiple genes (Supplementary Table S1). This distinction matters for their validity on targeted sequencing data.

Two of the tools follow the within-gene approach: 20/20+ and ActiveDriver. To sample from their null distributions, 20/20+ randomly relocates mutations within the gene, while ActiveDriver uses Poisson regression to model the background mutation rate within the gene of interest. Since both tools only consider mutations within each gene, their estimates will not depend on which other genes have been sequenced. This was confirmed by our results, which showed that identical results were obtained on targeted sequencing data when compared to whole-exome data. Therefore, these tools can be considered valid for targeted sequencing data driver detection.

OncodriveFML, OncodriveCLUSTL, MutSigCV, dNdSCv, and DriverML use an across-genes estimation of their null distribution. OncodriveFML and OncodriveCLUSTL randomly sample passenger mutations based on trinucleotide mutation probabilities estimated from mutations across all available genes. Similarly, MutSigCV and dNdScv estimate probabilities of passenger mutations based on their association with covariates such as gene length, replication time, or epigenetic marks across all available genes. Finally, DriverML samples passenger mutations from a Poisson distribution parameterized by a background mutation rate estimated from clusters of genes with similar genomic features. This dependence on mutations in other genes risks yielding different results when applied to targeted data as opposed to whole-exome data. Our results indicate that these tools’ driver predictions on targeted sequencing data do indeed differ from their predictions on whole-exome data. However, while this led to highly discordant predictions for MutSigCV and DriverML, the results for OncodriveFML, OncodriveCLUSTL, and dNdScv mostly resembled the whole-exome results.

A potential pitfall for tools that use across-genes estimation pertains to how they interpret the absence of mutations in genes that were not sequenced. Incorrectly assuming that those genes are never mutated has drastic consequences for estimates of passenger mutation rates. Background mutation rates may be underestimated, and as a consequence, evidence for positive selection may already be found at lower mutation frequencies. This was confirmed by our observation that genes identified as driver by DriverML and MutSigCV in the targeted mutation data only tend to have lower mutation frequencies. To alleviate this problem, dNdScv can be instructed to consider a selected list of panel genes only. MutSigCV and DriverML do not explicitly provide such an option. An interesting direction for future research on driver gene identification from targeted sequencing data is to explore how background models based on across-genes estimation can best be adapted to mutation data comprising only a small subset of genes.

When comparing identified driver genes with curated cancer genes from the Cancer Gene Census, we observed that for some cancer types and some tools, the targeted sequencing analyses recovered known cancer driver genes that were not identified in the whole-exome analyses and vice versa. Often, those genes had nominally significant *P*-values in both analyses, but multiple testing correction rendered them non-significant for one of the two. If instead, we focus on the genes that were not even nominally significant in either the whole-exome or targeted sequencing analyses, only differences obtained with MutSigCV or DriverML remain (Supplementary Tables S9 and S10). Notably, most of those genes have relatively low mutation frequencies in the corresponding cancer type. This may suggest that a high mutation frequency is such strong evidence for a driver gene that differences in background model estimation become less important. Yet with lower mutation frequencies, conclusions drawn based on the models may start to diverge.

It cannot be said with certainty whether driver genes only identified in either the whole-exome or targeted sequencing analyses are in fact false positives. Mutations in many of those genes have previously been linked to the cancer type for which our analyses classified them as drivers. For example, *NOTCH1* mutations in bladder cancer (Maraver *et al.* 2015), *RAC1* mutations in melanoma (Lodde *et al.* 2023), *FGFR2* mutations in endometrial cancer (Dixit *et al.* 2023), and *MSH2* and *MSH6* mutations in colon cancer (Haraldsdottir *et al.* 2014) were only identified as drivers in the whole-exome analyses. All have published evidence for their role in the corresponding cancer type. Likewise, among the genes identified as driver genes in the targeted sequencing analyses only, several were previously described as driver genes for the corresponding cancer type; e.g. *AKT1* in lung cancer (De Marco *et al.* 2015), *PTPRT* in head and neck cancer (Lui *et al.* 2014), *BRCA2* in ovarian cancer (Moschetta *et al.* 2016), and *ERBB4* in lung cancer (Kurppa *et al.* 2016). Nevertheless, it is important to recognize that MutSigCV and DriverML classified many additional genes as driver gene in the targeted sequencing analyses only. While a few are known driver genes, we expect that many will simply be false positives.

Our goal in this study was not to benchmark the performance of the tools for driver gene detection in general. All tools we tested have previously been evaluated in the context of whole-exome sequencing data, and all were found to perform well in that setting (Tokheim *et al.* 2016, Porta-Pardo *et al.* 2017). In this study, we aimed to assess their validity in a context they were not primarily designed for, i.e. analyzing mutation data obtained by targeted sequencing.

Many other tools for driver gene detection have been published in addition to the seven tools we evaluated, and many new methods continue to be published. In this work, we aimed for a selection of available tools representative of the statistical approaches used by a wider range of tools. Since statistical approaches and assumptions are often shared across tools, we expect that our conclusions will also be informative for evaluating the validity of tools we did not test. Moreover, we suggest using our analytical workflow when considering other tools for targeted genome sequencing data.

We presented a systematic approach to assessing the validity of driver detection tools for targeted genome sequencing data. This approach can be applied to existing tools or during algorithm development. Our results showed that tools based on a within-gene null distribution produce identical results on targeted sequencing data and whole-exome data. This was not the case for tools employing an across-genes null distribution, yet several of those still produced usable results for targeted sequencing data. Based on our results, 20/20+, ActiveDriver, OncodriveFML, OncodriveCLUSTL, and dNdScv are valid for driver gene identification on targeted genome sequencing data, whereas MutSigCV and DriverML should be avoided in that context.

## Supplementary Material

vbae073_Supplementary_Data

## Data Availability

All datasets referenced in the Methods section are publicly available from TCGA. Mutation dataset as a MAF file (MC3v0.2.8) as well as clinical metadata for all samples (clinical_PANCAN_patient_with_followup) were extracted from the publicly available TCGA PanCanAtlas supplemental data: https://gdc.cancer.gov/about-data/publications/pancanatlas. R scripts used for the analyses are available on GitHub: https://github.com/SchmidtGroupNKI/TGSdrivergene_validity.
