# Supplementary material for: Assessing the validity of driver gene identification tools for targeted genome sequencing data
Source: Bioinform Adv. 2024 May 23;4(1):vbae073. doi: 10.1093/bioadv/vbae073 (PMC11132814; doi:10.1093/bioadv/vbae073)
Supplement: vbae073_Supplementary_Data [file vbae073_supplementary_data.pdf]

SUPPLEMENTARY MATERIAL

Assessing the validity of driver gene identification tools for targeted genome sequencing data

F. Rojas-Rodriguez[1], M.K. Schmidt[1,2], S. Canisius[1,3].

[1] Division of Molecular Pathology, The Netherlands Cancer Institute – Antoni van Leeuwenhoek Hospital, Amsterdam, The Netherlands.

[2] Department of Clinical Genetics, Leiden University Medical Center, Leiden, The Netherlands.

[3] Division of Molecular Carcinogenesis, The Netherlands Cancer Institute - Antoni van Leeuwenhoek Hospital, Amsterdam, The Netherlands.

Corresponding author:

Sander Canisius

Division of Molecular Pathology, the Netherlands Cancer Institute – Antoni van Leeuwenhoek Hospital, Amsterdam, The Netherlands.

Division of Molecular Carcinogenesis, The Netherlands Cancer Institute - Antoni van Leeuwenhoek Hospital, Amsterdam, The Netherlands.

Plesmanlaan 121, 1066 CX Amsterdam

Contact information: s.canisius@nki.nl

SUPPLEMENTARY FIGURES

Supplementary figure 1. Mutational frequency and sample size across all major cancer types in TCGA.

Supplementary figure 2. Concordance of p-values between whole-exome and targeted datasets.

Supplementary figure 3. Comparison of the lengths of genes identified exclusively in the targeted sequencing or whole-exome datasets, or in both.

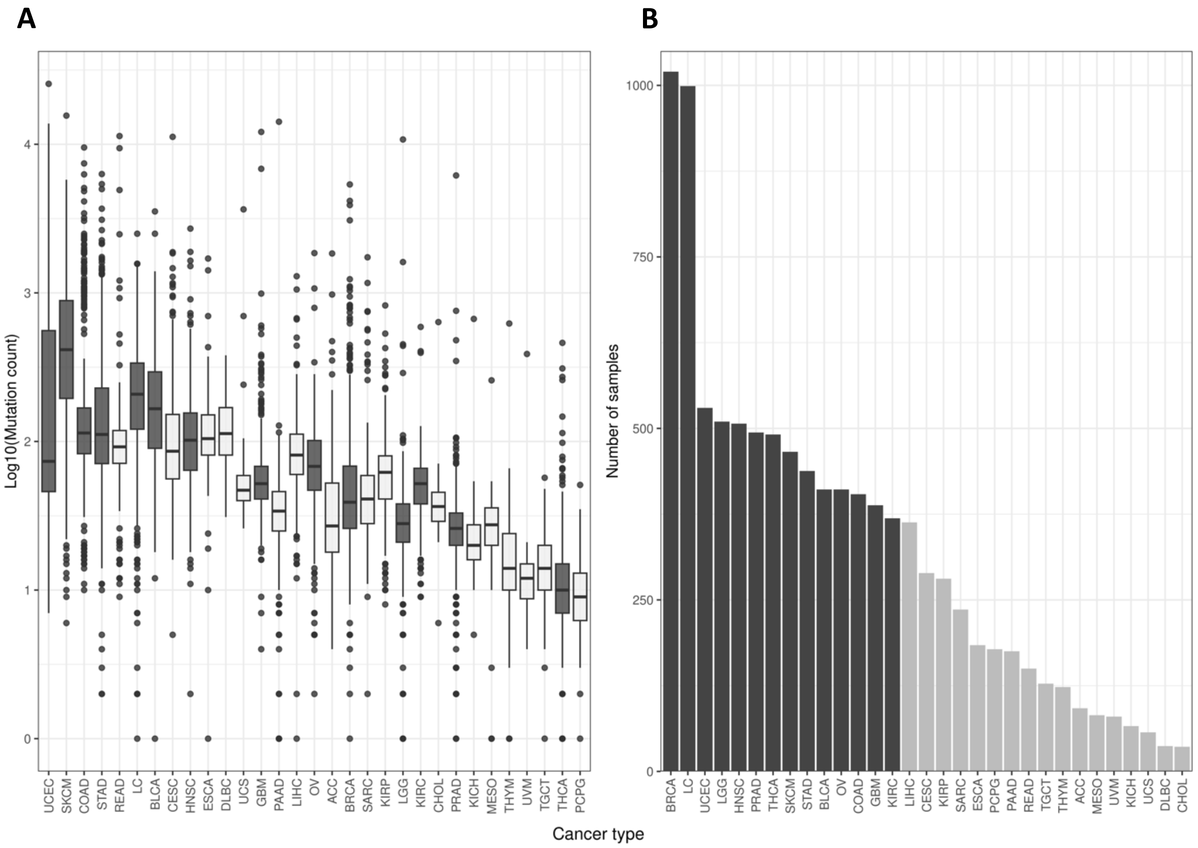

Supplementary figure 1. Mutation counts per tumor (A) and sample size (B) across all cancer types in TCGA. Cancer types selected for the current analysis are highlighted in black. The available and (in bold) included cancer types are adrenocortical carcinoma (ACC), bladder urothelial carcinoma (**BLCA**), breast invasive carcinoma (**BRCA**), cervical squamous cell carcinoma and endocervical adenocarcinoma (CESC), cholangiocarcinoma (CHOL), colon adenocarcinoma (**COAD**), lymphoid neoplasm diffuse large B-cell lymphoma (DLBC), esophageal carcinoma (ESCA), glioblastoma multiforme (**GBM**), head and neck squamous cell carcinoma (**HNSC**), kidney chromophobe (KICH), kidney renal clear cell carcinoma (**KIRC**), kidney renal papillary cell carcinoma (KIRP), Lung adenocarcinoma and lung squamous cell carcinoma cancer (LUAD and LUSC respectively, referred to as lung cancer (**LC**) in the main text), brain lower grade glioma (**LGG**), liver hepatocellular carcinoma (LIHC), mesothelioma (MESO), ovarian serous cystadenocarcinoma (**OV**), pancreatic adenocarcinoma (PAAD), pheochromocytoma and paraganglioma (PCPG), prostate adenocarcinoma (**PRAD**), rectum adenocarcinoma (READ), sarcoma (SARC), skin cutaneous melanoma (**SKCM**), stomach adenocarcinoma (**STAD**), testicular germ cell tumors (TGCT), thyroid carcinoma (**THCA**), thymoma (THYM), uterine corpus endometrial carcinoma (**UCEC**), uterine carcinosarcoma (UCS) and uveal melanoma (UVM).

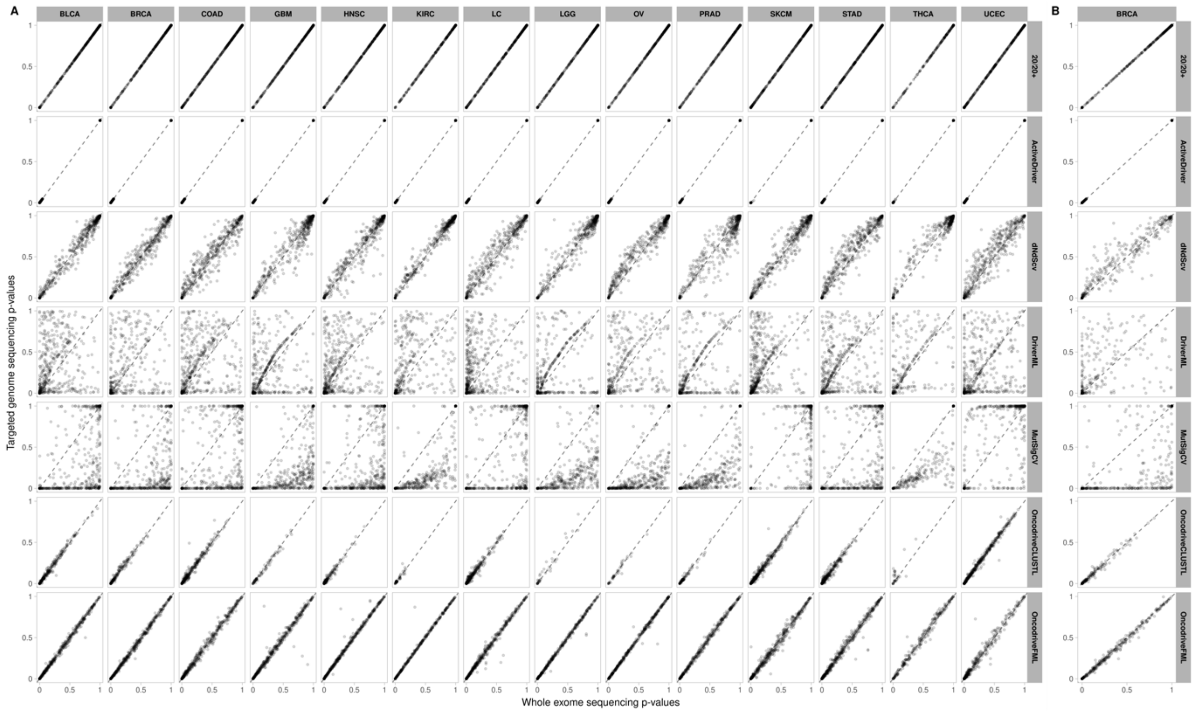

Supplementary figure 2. Concordance of p-values between whole-exome and targeted datasets. The tools used (rows) were 20/20+, ActiveDriver, dNdScv, DriverML, MutSigCV, OncodriveCLUSTL and OncodriveFML. (A) Results based on the MSK-IMPACT gene panel to obtain the targeted genome sequencing datasets across all cancer types and (B) using the B-CAST gene panel only for the breast cancer dataset. Scatter plots showing a near perfect concordance between whole-exome and targeted p-values were considered to perform well in the context of targeted genome sequencing. The dashed diagonal line indicates optimal concordance.

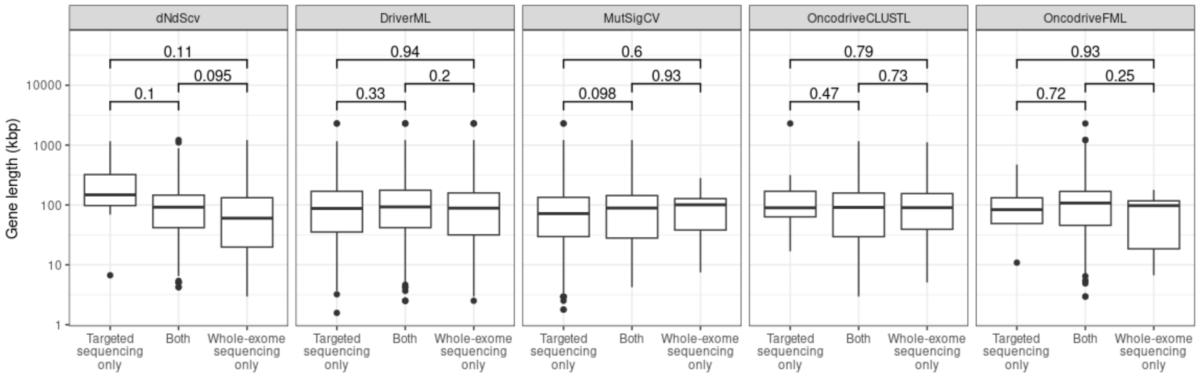

Supplementary figure 3. Comparison of the lengths of genes identified exclusively in the targeted sequencing or whole-exome datasets, or in both. Results for all 14 cancer types were aggregated. P values were obtained with the Wilcoxon rank sum test. ActiveDriver and 20/20+ were not included because the exact same sets of genes were identified in the targeted sequencing dataset and in the whole-exome dataset.

SUPPLEMENTARY TABLES

Supplementary table 1. Overview of the statistical methods and their null distributions for the seven driver gene identification tools.

Supplementary table 2. Mapping of TCGA cancer type acronyms to tumor types used in the Cancer Gene Census annotation.

Supplementary table 3. Mean absolute error of p-values based on targeted genome sequencing.

Supplementary table 4. False discovery rate and sensitivity using whole-exome as reference. Results based on a Benjamini-Hochberg multiple testing correction threshold of 0.01.

Supplementary table 5. False discovery rate and sensitivity using whole-exome as reference. Results based on a Benjamini-Hochberg multiple testing correction threshold of 0.05.

Supplementary table 6. Numbers of driver genes identified in the breast cancer data using the MSK-IMPACT panel, the B-CAST panel, or both.

Supplementary table 7. Numbers of known driver genes (according to the Cancer Gene Census) identified in the targeted data but not in the whole-exome data.

Supplementary table 8. Numbers of known driver genes (according to the Cancer Gene Census) identified in the whole-exome data but not in the targeted data.

Supplementary table 9. Known driver genes (according to the Cancer Gene Census) identified in the whole-exome data but not in the targeted data.

Supplementary table 10. Known driver genes (according to the Cancer Gene Census) identified in the targeted data but not in the whole-exome data.

Supplementary table 1. Overview of the statistical methods and their null distributions for the seven driver gene identification tools. The type of null distribution indicates whether the null distribution estimated for a gene uses only mutations targeting that gene (within-gene) or also mutations targeting other genes (across-genes). The rationale for assigning a method to its listed type is printed in bold in the description column.

Tool	Type of null distribution	Description of the statistical model and its null distribution
20/20+	Within-gene	20/20+ (Tokheim, et al., 2016) performs two steps to test the driver status of a gene: first it computes cancer driver scores for oncogenes, tumor suppressors and passengers; then it estimates the statistical significance of those driver scores. To obtain the driver scores, a random forest method is applied to predict the oncogene, tumor suppressor or passenger status for each gene independently using a set of 24 features (e.g., missense to silent mutation ratio, functional impact scores). The random forest model is trained on an external dataset using all drivers obtained by the 20/20 rule method (Vogelstein, et al., 2013). Each gene is scored as the fraction of trees classifying a gene as a driver. P-values are obtained by sampling from the null distribution with Monte Carlo simulation. **A fixed number of simulated mutations, equal to the observed mutations in each gene, are moved along the gene with uniform probability while maintaining the original nucleotide context.** Each simulated nucleotide change is then scored using the same random forest approach described previously. The p-value for each gene is computed as the fraction of the simulated scores equal to or greater than the score obtained from the observed mutations.
ActiveDriver	Within-gene	ActiveDriver (Reimand and Bader, 2013) identifies driver genes using signals of positive selection on post-translational modification sites. The model's null hypothesis states that there is no difference in mutational rate between phosphorylation sites and the rest of the gene. ActiveDriver then tests whether a phosphosite region is mutated more frequently than the average mutation rate of the gene containing the mutation. To do so, missense mutation counts for each gene are assumed to follow a Poisson distribution. This leads to a Poisson regression model with the mutation counts per residue in the protein sequence as dependent variable. **For each phosphosite, two models are compared: an intercept-only null model, which assumes an equal mutation rate all across the gene; and an alternative model with additional covariates that can capture a different mutation rate for phosphosites.** P-values for each individual phosphorylation site are obtained by comparing the null model to the alternative model using the deviance statistic. Subsequently, p-values for each gene are obtained as a product of the significant phosphorylation site's p-values contained in the gene.
dNdScv	Across-genes	dNdScv (Martincorena, et al., 2017) dNdScv is an extension of the dN/dS model for detecting positive or negative selection in mutation data. The latter is based on the ratio of the non-synonymous substitution rate and the synonymous substitution rate, where a deviation from 1 indicates selection. A formulation of this model in terms of a Poisson distribution enables estimating the dN/dS ratio corrected for the effects of different types of substitution and sequence composition. Whereas previous applications of dN/dS assumed a single background substitution rate across all genes, dNdScv extends the methodology by modeling varying mutation rates across genes. To do so, it assumes that the background substitution rate for a gene follows a Gamma distribution parameterized by the different types of substitution, sequence composition, and additional covariates that describe epigenetic marks within the gene region. Combining this with the original dN/dS Poisson model leads to a Gamma-Poisson mixture for which the parameters can be estimated from mutation data across all genes using negative binomial regression. As a result, **gene-specific estimates of the background substitution rate borrow information from genes that are similar based on their (epi-)genomic characteristics**. The p-value for a gene is computed using the likelihood ratio test where the null model based on a dN/dS ratio fixed to 1 is compared to the maximum likelihood estimate of an unrestricted model.
DriverML	Across-genes	DriverML (Han, et al., 2019) combines a weighted Rao's score test and a machine learning model to estimate the driver status of a gene. The model assumes that the silent and non-silent mutations in the data follow a Poisson distribution. The Poisson distributions for both silent and non-silent mutations are parameterized by a gene's background mutation rate. **To make the estimates of these background mutation rates more robust, they are not computed for each individual gene, but for clusters of genes.** Genes are

		clustered based on covariates (genomic context, expression level, etc.), after which each cluster's background mutational rate is computed by an empirical Bayes method. Rao's score statistic is used to test whether silent and non-silent mutations have different mutation rates, separately for five non-silent mutation types. A global weighted score statistic is obtained by combining the scores of all mutation types, weighted by functional impact scores. The null distribution of the global score statistic is obtained by Monte Carlo simulation. The global Rao score for each gene is then compared to the global scores from the Monte Carlo simulations. P-values are obtained based on the number of simulated scores that are equal to or greater than the observed score.
MutSigCV	Across-genes	MutSigCV (Lawrence, et al., 2013) computes gene scores that contrast observed mutation counts against tumor- and gene-specific background mutation rates, and prioritizes genes mutated across many tumors over genes with multiple mutations in the same tumor. **The estimates of a gene's background mutation rate use the frequency of non-coding and synonymous mutations in that gene and in a selected number of most similar genes**, where similarity is determined based on gene characteristics such as expression level and replication time. Each gene is placed in a high-dimensional covariate space based on its gene characteristics. The closest genes in the covariate space are selected, after which the background mutation rate is computed across all selected genes. For assessing significance, an analytical null distribution has been derived for the gene scores based on closed form probabilities for the individual components of the gene score. A gene's p-value corresponds to the probability of a gene score equal to or greater than the observed score according to the null distribution.
OncodriveCLUSTL	Across-genes	OncodriveCLUSTL (Arnedo-Pac, et al., 2019) detects driver genes by testing for non-random patterns of mutational clustering across a gene region. For each gene, it first identifies one or more clusters corresponding to high regional densities of mutations. Once these clusters are obtained, each one is scored based on the number of mutations and their distribution. The score for each gene is equal to the sum of all cluster scores within the gene. The null distribution of the gene score is obtained by Monte Carlo simulation. Random sets of mutations, of size equal to the number of observed mutations in the gene, are sampled. **The probability of a random mutation at a specific location is conditioned on the local nucleotide context and is estimated from mutations across all genes and tumors.** For each random set of mutations, the clustering-based gene score is computed. Based on these samples from the null distribution, three types of p-value are obtained: an empirical p-value corresponding to the fraction of simulations with a gene score greater or equal to the observed score; an analytical p-value based on a gaussian kernel density estimate fitted to the simulated gene scores; and a second analytical p-value based on a gaussian kernel density estimate fitted to the top-scoring cluster only.
OncodriveFML	Across-genes	OncodriveFML (Mularoni, et al., 2016) makes use of a functional impact score and mutational signatures to estimate the driver status of a gene. Gene-level summary scores are computed as the mean functional impact score of mutations targeting the gene across all tumors. This functional impact score is based on the CADD scoring system (Kircher, et al., 2014). The null distribution of these gene scores is obtained by Monte Carlo simulation. **Groups of random nucleotide changes targeting a gene are sampled with a probability that is conditioned on mutational signatures computed from the observed mutations across all genes and tumors.** The same approach used to compute functional impact scores for the observed mutations is also used for the random nucleotide changes. Finally, empirical p-values are computed based on the fraction of random functional impact scores that are greater than or equal to the observed functional impact score for each gene.

Supplementary table 2. Mapping of TCGA cancer type acronyms to tumor types used in the Cancer Gene Census annotation. We used this mapping to compose cancer type-specific sets of known driver genes.

TCGA acronym	CGC tumor types
BLCA	"bladder", "bladder carcinoma"
BRCA	"breast", "breast cancer", "secretory breast", "breast carcinoma", "lobular breast", "luminal A breast"
COAD	"colorectal", "colorectal cancer", "colorectal carcinoma", "colon", "colon carcinoma", "colorectal adenocarcinoma"
GBM	"glioblastoma", "paediatric glioblastoma"
HNSC	"HNSCC", "head and neck", "head and neck SCC", "head and neck cancer", "head-neck squamous cell"
KIRC	"renal", "RCC", "clear cell renal carcinoma", "renal cell carcinoma"
LC	"NSCLC", "lung", "SCC", "lung adenocarcinoma", "lung cancer", "small cell lung carcinoma", "lung SCC", "lung carcinoma"
LGG	"DIPG", "glioma"
OV	"ovarian", "clear cell ovarian carcinoma", "ovarian cancer", "serous ovarian", "epithelial ovarian", "ovarian carcinoma", "ovarian mixed germ cell tumour", "ovary"
PRAD	"prostate", "prostate cancer", "prostate carcinoma"
SKCM	"melanoma", "skin", "skin cancer"
STAD	"gastric", "gastric cancer", "stomach carcinoma", "gastric carcinoma"
THCA	"papillary thyroid", "follicular thyroid", "thyroid", "thyroid cancer"
UCEC	"endometrial", "endometrial carcinoma", "endometrial stromal sarcoma", "endometrioid carcinoma", "endometrial stromal tumour", "endometrium", "uterine serous carcinoma"

Supplementary table 3. Mean absolute error of p-values based on targeted genome sequencing. The results from the whole-exome dataset were used as reference for each cancer type independently. All 14 cancer types were analyzed using the MSK-IMPACT gene panel. In addition, breast cancer (BRCA) was also analyzed using the B-CAST gene panel.

**Tool**	**MSK-IMPACT**														**B-CAST**
	**BLCA**	**BRCA**	**COAD**	**GBM**	**HNSC**	**KIRC**	**LC**	**LGG**	**OV**	**PRAD**	**SKCM**	**STAD**	**THCA**	**UCEC**	**BRCA**
20/20+	5.9e-07	4.1e-07	3.8e-07	0	8.5e-08	0	9.5e-07	0	0	0	2.8e-06	1.6e-06	0	2.6e-12	1.1e-10
ActiveDriver	0	0	0	0	0	0	2.4e-05	0	0	0	1.4e-04	0	0	0	0
dNdScv	4e-02	4.7e-02	7.1e-02	5.9e-02	5.4e-02	3e-02	7e-02	4.5e-02	6.6e-02	7.3e-02	5e-02	8.2e-02	5e-02	8.6e-02	7.4e-02
DriverML	2.5e-01	2e-01	2.4e-01	2.1e-01	2.3e-01	2.6e-01	2.5e-01	2e-01	2e-01	2e-01	2.3e-01	2.2e-01	2e-01	2.1e-01	1.6e-01
MutSigCV	3.5e-01	4e-01	3.5e-01	3.9e-01	4.3e-01	2.3e-01	3e-01	2.9e-01	3.1e-01	2.4e-01	1.9e-01	3.3e-01	1.2e-01	1.9e-01	3.7e-01
OncodriveCLUSTL	1.4e-02	1.4e-02	1.7e-02	1.5e-02	9.1e-03	9.6e-03	1.3e-02	3.3e-02	1.4e-02	1.1e-02	1.6e-02	1.4e-02	5.3e-02	1.4e-02	1.4e-02
OncodriveFML	1.2e-02	1.3e-02	1.7e-02	2.1e-02	1.2e-02	8.1e-03	1.6e-02	9.2e-03	9.2e-03	1.4e-02	2.3e-02	1.9e-02	2.5e-02	1.8e-02	1.6e-02

BLCA: bladder urothelial carcinoma. BRCA: breast invasive carcinoma. COAD: colon adenocarcinoma. GBM: glioblastoma multiforme. HNSC: head and neck squamous cell carcinoma. KIRC: kidney renal clear cell carcinoma. LC: lung adenocarcinoma and lung squamous cell carcinoma combined. LGG: brain lower grade glioma. OV: ovarian serous cystadenocarcinoma. PRAD: prostate adenocarcinoma. SKCM: skin cutaneous melanoma. STAD: stomach adenocarcinoma. THCA: thyroid carcinoma. UCEC: uterine corpus endometrial carcinoma.

Supplementary table 4. False discovery rate and sensitivity using whole-exome as reference. 95% confidence intervals are reported in parentheses. Both metrics were computed after detecting driver genes using Benjamini-Hochberg multiple testing correction thresholds of 0.01. Cells are empty if no driver genes were found to be significant. All 14 cancer types were analyzed using the MSK-IMPACT gene panel. In addition, breast cancer (BRCA) was also analyzed using the B-CAST gene panel.

		MSK-IMPACT														B-CAST
**Tools**		**BLCA**	**BRCA**	**COAD**	**GBM**	**HNSC**	**KIRC**	**LC**	**LGG**	**OV**	**PRAD**	**SKCM**	**STAD**	**THCA**	**UCEC**	**BRCA**
20/20+	False discovery rate	0 (0 - 0.2)	0 (0 - 0.31)	0 (0 - 0.23)	0 (0 - 0.41)	0 (0 - 0.28)	0 (0 - 0.84)	0 (0 - 0.2)	0 (0 - 0.41)	0 (0 - 0.84)	0 (0 - 0.46)	0 (0 - 0.12)	0 (0 - 0.28)	0 (0 - 0.71)	0 (0 - 0.09)	0 (0 - 0.28)
ActiveDriver		0 (0 - 0.11)	0 (0 - 0.21)	0 (0 - 0.07)	0 (0 - 0.13)	0 (0 - 0.11)	0 (0 - 0.52)	0.02 (0 - 0.11)	0 (0 - 0.14)	0 (0 - 0.34)	0 (0 - 0.22)	0 (0 - 0.04)	0 (0 - 0.09)	0 (0 - 0.37)	0 (0 - 0.05)	0 (0 - 0.13)
dNdScv		0 (0 - 0.1)	0 (0 - 0.14)	0 (0 - 0.15)	0.08 (0 - 0.38)	0 (0 - 0.12)	0 (0 - 0.28)	0.04 (0 - 0.18)	0.11 (0.01 - 0.35)	0 (0 - 0.52)	0 (0 - 0.23)	0.1 (0 - 0.45)	0.05 (0 - 0.25)	0 (0 - 0.52)	0.03 (0 - 0.16)	0 (0 - 0.12)
DriverML		0.09 (0.01 - 0.28)	0.35 (0.28 - 0.43)	0.2 (0.06 - 0.44)	0.62 (0.42 - 0.79)	0.61 (0.45 - 0.75)	0.5 (0.35 - 0.65)	0.46 (0.32 - 0.61)	0.53 (0.36 - 0.69)	0.11 (0.06 - 0.19)	0.57 (0.41 - 0.71)	0.2 (0.01 - 0.72)	0.51 (0.34 - 0.68)	0.25 (0.07 - 0.52)	0.28 (0.15 - 0.45)	0.17 (0.11 - 0.25)
MutSigCV		0.84 (0.77 - 0.9)	0.85 (0.79 - 0.9)	0.84 (0.77 - 0.9)	0.78 (0.64 - 0.88)	0.8 (0.71 - 0.87)	0.68 (0.49 - 0.83)	0.8 (0.72 - 0.87)	0.72 (0.56 - 0.85)	0.92 (0.83 - 0.97)	0.69 (0.52 - 0.84)	0.76 (0.58 - 0.89)	0.84 (0.77 - 0.9)	0.44 (0.14 - 0.79)	0.57 (0.42 - 0.71)	0.82 (0.76 - 0.88)
OncodriveCLUSTL		0.08 (0 - 0.38)	0.29 (0.04 - 0.71)	0.22 (0.03 - 0.6)	0.33 (0.01 - 0.91)	0.1 (0.02 - 0.27)		0.17 (0.04 - 0.41)	0 (0 - 0.71)	0 (0 - 0.98)	0.33 (0.01 - 0.91)	0.29 (0.1 - 0.56)	0.15 (0.03 - 0.38)	0 (0 - 0.71)	0.12 (0.03 - 0.32)	0.33 (0.07 - 0.7)
OncodriveFML		0.03 (0 - 0.18)	0 (0 - 0.15)	0 (0 - 0.11)	0.14 (0 - 0.58)	0 (0 - 0.15)	0 (0 - 0.34)	0 (0 - 0.11)	0 (0 - 0.26)	0 (0 - 0.6)	0 (0 - 0.6)	0.04 (0 - 0.2)	0 (0 - 0.19)	0 (0 - 0.98)	0.01 (0 - 0.05)	0.12 (0.02 - 0.3)
20/20+	Sensitivity	1 (0.8 - 1)	1 (0.69 - 1)	1 (0.77 - 1)	1 (0.59 - 1)	1 (0.72 - 1)	1 (0.16 - 1)	1 (0.8 - 1)	1 (0.59 - 1)	1 (0.16 - 1)	1 (0.54 - 1)	1 (0.88 - 1)	1 (0.72 - 1)	1 (0.29 - 1)	1 (0.91 - 1)	1 (0.72 - 1)
ActiveDriver		1 (0.89 - 1)	1 (0.79 - 1)	1 (0.93 - 1)	1 (0.87 - 1)	1 (0.89 - 1)	1 (0.48 - 1)	1 (0.92 - 1)	1 (0.86 - 1)	1 (0.66 - 1)	1 (0.78 - 1)	1 (0.96 - 1)	1 (0.91 - 1)	1 (0.63 - 1)	1 (0.95 - 1)	1 (0.87 - 1)
dNdScv		0.88 (0.73 - 0.96)	0.93 (0.76 - 0.99)	0.85 (0.66 - 0.96)	0.85 (0.55 - 0.98)	0.93 (0.78 - 0.99)	0.92 (0.62 - 1)	0.82 (0.65 - 0.93)	0.94 (0.71 - 1)	1 (0.48 - 1)	0.93 (0.68 - 1)	1 (0.66 - 1)	0.83 (0.61 - 0.95)	0.83 (0.36 - 1)	0.82 (0.66 - 0.92)	0.88 (0.71 - 0.96)
DriverML		0.38 (0.25 - 0.52)	0.71 (0.63 - 0.77)	0.25 (0.15 - 0.37)	0.26 (0.14 - 0.42)	0.6 (0.41 - 0.77)	0.71 (0.52 - 0.86)	0.9 (0.74 - 0.98)	0.22 (0.14 - 0.33)	0.78 (0.7 - 0.85)	0.32 (0.21 - 0.45)	0.33 (0.1 - 0.65)	0.45 (0.29 - 0.62)	0.4 (0.23 - 0.59)	0.27 (0.19 - 0.37)	0.72 (0.63 - 0.79)
MutSigCV		0.92 (0.75 - 0.99)	1 (0.86 - 1)	0.95 (0.75 - 1)	0.92 (0.62 - 1)	0.96 (0.78 - 1)	1 (0.69 - 1)	0.96 (0.8 - 1)	0.92 (0.64 - 1)	1 (0.48 - 1)	1 (0.72 - 1)	0.89 (0.52 - 1)	0.91 (0.72 - 0.99)	1 (0.48 - 1)	0.79 (0.59 - 0.92)	1 (0.87 - 1)
OncodriveCLUSTL		0.92 (0.62 - 1)	0.71 (0.29 - 0.96)	1 (0.59 - 1)	1 (0.16 - 1)	0.54 (0.39 - 0.68)		0.71 (0.48 - 0.89)	0.6 (0.15 - 0.95)	1 (0.03 - 1)	1 (0.16 - 1)	1 (0.74 - 1)	0.61 (0.41 - 0.78)	1 (0.29 - 1)	0.95 (0.77 - 1)	1 (0.54 - 1)
OncodriveFML		0.97 (0.82 - 1)	1 (0.85 - 1)	1 (0.89 - 1)	1 (0.54 - 1)	0.96 (0.79 - 1)	1 (0.66 - 1)	0.91 (0.77 - 0.98)	1 (0.74 - 1)	1 (0.4 - 1)	1 (0.4 - 1)	0.96 (0.8 - 1)	1 (0.81 - 1)	1 (0.03 - 1)	0.98 (0.94 - 1)	1 (0.85 - 1)

BLCA: bladder urothelial carcinoma. BRCA: breast invasive carcinoma. COAD: colon adenocarcinoma. GBM: glioblastoma multiforme. HNSC: head and neck squamous cell carcinoma. KIRC: kidney renal clear cell carcinoma. LC: lung adenocarcinoma and lung squamous cell carcinoma combined. LGG: brain lower grade glioma. OV: ovarian serous cystadenocarcinoma. PRAD: prostate adenocarcinoma. SKCM: skin cutaneous melanoma. STAD: stomach adenocarcinoma. THCA: thyroid carcinoma. UCEC: uterine corpus endometrial carcinoma.

Supplementary table 5. False discovery rate and sensitivity using whole-exome as reference. 95% confidence intervals are reported in parentheses. Both metrics were computed after detecting driver genes using Benjamini-Hochberg multiple testing correction thresholds of 0.05. All 14 cancer types were analyzed using the MSK-IMPACT gene panel. In addition, breast cancer (BRCA) was also analyzed using the B-CAST gene panel.

		MSK-IMPACT														B-CAST
**Tools**		**BLCA**	**BRCA**	**COAD**	**GBM**	**HNSC**	**KIRC**	**LC**	**LGG**	**OV**	**PRAD**	**SKCM**	**STAD**	**THCA**	**UCEC**	**BRCA**
20/20+	False discovery rate	0 (0 - 0.18)	0 (0 - 0.21)	0 (0 - 0.21)	0 (0 - 0.37)	0 (0 - 0.23)	0 (0 - 0.71)	0 (0 - 0.15)	0 (0 - 0.37)	0 (0 - 0.84)	0 (0 - 0.37)	0 (0 - 0.09)	0 (0 - 0.26)	0 (0 - 0.6)	0 (0 - 0.08)	0 (0 - 0.21)
ActiveDriver		0 (0 - 0.07)	0 (0 - 0.07)	0 (0 - 0.05)	0 (0 - 0.08)	0 (0 - 0.05)	0 (0 - 0.26)	0 (0 - 0.04)	0 (0 - 0.09)	0 (0 - 0.15)	0 (0 - 0.09)	0 (0 - 0.04)	0 (0 - 0.06)	0 (0 - 0.19)	0 (0 - 0.03)	0 (0 - 0.08)
dNdScv		0 (0 - 0.09)	0.03 (0 - 0.18)	0 (0 - 0.14)	0 (0 - 0.23)	0 (0 - 0.11)	0.08 (0 - 0.36)	0.03 (0 - 0.16)	0.1 (0.01 - 0.3)	0 (0 - 0.52)	0.06 (0 - 0.3)	0.09 (0 - 0.41)	0 (0 - 0.14)	0 (0 - 0.46)	0 (0 - 0.1)	0 (0 - 0.11)
DriverML		0.17 (0.08 - 0.31)	0.22 (0.16 - 0.28)	0.29 (0.15 - 0.46)	0.62 (0.53 - 0.71)	0.38 (0.26 - 0.51)	0.45 (0.32 - 0.6)	0.43 (0.32 - 0.55)	0.49 (0.41 - 0.57)	0.1 (0.05 - 0.17)	0.56 (0.47 - 0.65)	0.14 (0 - 0.58)	0.25 (0.13 - 0.4)	0.31 (0.14 - 0.52)	0.24 (0.13 - 0.39)	0.11 (0.06 - 0.18)
MutSigCV		0.85 (0.79 - 0.9)	0.87 (0.82 - 0.91)	0.85 (0.78 - 0.9)	0.88 (0.79 - 0.93)	0.87 (0.81 - 0.92)	0.85 (0.74 - 0.92)	0.82 (0.75 - 0.88)	0.79 (0.68 - 0.88)	0.95 (0.9 - 0.98)	0.82 (0.71 - 0.9)	0.82 (0.67 - 0.92)	0.86 (0.8 - 0.91)	0.64 (0.35 - 0.87)	0.58 (0.45 - 0.71)	0.83 (0.76 - 0.88)
OncodriveCLUSTL		0.29 (0.11 - 0.52)	0 (0 - 0.13)	0.26 (0.09 - 0.51)	0.33 (0.01 - 0.91)	0.07 (0.03 - 0.15)	0.03 (0 - 0.14)	0.08 (0.03 - 0.16)	0 (0 - 0.37)	0 (0 - 0.6)	0 (0 - 0.25)	0.04 (0 - 0.2)	0.05 (0.01 - 0.13)	0 (0 - 0.52)	0.09 (0.02 - 0.24)	0.03 (0 - 0.18)
OncodriveFML		0 (0 - 0.09)	0 (0 - 0.11)	0.07 (0.02 - 0.16)	0 (0 - 0.37)	0 (0 - 0.11)	0 (0 - 0.34)	0.02 (0 - 0.11)	0 (0 - 0.22)	0 (0 - 0.6)	0 (0 - 0.6)	0.08 (0.02 - 0.21)	0.07 (0.01 - 0.23)	0 (0 - 0.84)	0.04 (0.02 - 0.08)	0.07 (0.01 - 0.19)
20/20+	Sensitivity	1 (0.82 - 1)	1 (0.79 - 1)	1 (0.79 - 1)	1 (0.63 - 1)	1 (0.77 - 1)	1 (0.29 - 1)	1 (0.85 - 1)	1 (0.63 - 1)	1 (0.16 - 1)	1 (0.63 - 1)	1 (0.91 - 1)	1 (0.74 - 1)	1 (0.4 - 1)	1 (0.92 - 1)	1 (0.79 - 1)
ActiveDriver		1 (0.93 - 1)	1 (0.93 - 1)	1 (0.95 - 1)	1 (0.92 - 1)	1 (0.95 - 1)	1 (0.74 - 1)	1 (0.96 - 1)	1 (0.91 - 1)	1 (0.85 - 1)	1 (0.91 - 1)	1 (0.96 - 1)	1 (0.94 - 1)	1 (0.81 - 1)	1 (0.97 - 1)	1 (0.92 - 1)
dNdScv		0.87 (0.73 - 0.95)	0.93 (0.78 - 0.99)	0.78 (0.6 - 0.91)	1 (0.77 - 1)	0.94 (0.8 - 0.99)	1 (0.74 - 1)	0.76 (0.6 - 0.88)	0.9 (0.7 - 0.99)	1 (0.48 - 1)	1 (0.78 - 1)	0.59 (0.33 - 0.82)	0.89 (0.71 - 0.98)	0.67 (0.3 - 0.93)	0.8 (0.65 - 0.9)	0.86 (0.71 - 0.95)
DriverML		0.49 (0.37 - 0.6)	0.77 (0.7 - 0.82)	0.34 (0.23 - 0.46)	0.66 (0.53 - 0.77)	0.59 (0.46 - 0.71)	0.71 (0.54 - 0.84)	0.43 (0.33 - 0.53)	0.68 (0.58 - 0.76)	0.75 (0.67 - 0.82)	0.76 (0.65 - 0.85)	0.11 (0.04 - 0.22)	0.48 (0.36 - 0.6)	0.43 (0.28 - 0.59)	0.28 (0.2 - 0.36)	0.72 (0.65 - 0.79)
MutSigCV		0.9 (0.74 - 0.98)	1 (0.87 - 1)	0.88 (0.7 - 0.98)	1 (0.74 - 1)	0.96 (0.78 - 1)	1 (0.69 - 1)	0.96 (0.8 - 1)	1 (0.78 - 1)	1 (0.59 - 1)	1 (0.74 - 1)	0.8 (0.44 - 0.97)	0.96 (0.79 - 1)	1 (0.48 - 1)	0.78 (0.6 - 0.91)	1 (0.89 - 1)
OncodriveCLUSTL		1 (0.78 - 1)	0.93 (0.77 - 0.99)	0.93 (0.68 - 1)	1 (0.16 - 1)	0.9 (0.82 - 0.95)	1 (0.91 - 1)	0.92 (0.84 - 0.97)	0.89 (0.52 - 1)	0.8 (0.28 - 0.99)	1 (0.75 - 1)	0.92 (0.75 - 0.99)	0.91 (0.83 - 0.96)	0.71 (0.29 - 0.96)	0.97 (0.83 - 1)	0.82 (0.65 - 0.93)
OncodriveFML		0.95 (0.83 - 0.99)	0.97 (0.85 - 1)	0.95 (0.86 - 0.99)	1 (0.63 - 1)	1 (0.89 - 1)	1 (0.66 - 1)	0.96 (0.86 - 0.99)	1 (0.78 - 1)	1 (0.4 - 1)	0.8 (0.28 - 0.99)	1 (0.9 - 1)	1 (0.87 - 1)	0.5 (0.07 - 0.93)	0.98 (0.95 - 1)	1 (0.91 - 1)

BLCA: bladder urothelial carcinoma. BRCA: breast invasive carcinoma. COAD: colon adenocarcinoma. GBM: glioblastoma multiforme. HNSC: head and neck squamous cell carcinoma. KIRC: kidney renal clear cell carcinoma. LC: lung adenocarcinoma and lung squamous cell carcinoma combined. LGG: brain lower grade glioma. OV: ovarian serous cystadenocarcinoma. PRAD: prostate adenocarcinoma. SKCM: skin cutaneous melanoma. STAD: stomach adenocarcinoma. THCA: thyroid carcinoma. UCEC: uterine corpus endometrial carcinoma. BH-FDR: Benjamini-Hochberg multiple testing correction threshold.

Supplementary table 6. Numbers of driver genes identified in the breast cancer data using the MSK-IMPACT panel, the B-CAST panel, or both.

Tool	MSK-IMPACT	B-CAST	Both
20/20+	14	14	14
ActiveDriver	9	9	9
dNdScv	28	26	26
DriverML	82	74	72
MutSigCV	75	80	75
OncodriveCLUSTL	8	9	7
OncodriveFML	26	26	26

Supplementary table 7. Numbers of known driver genes (according to the Cancer Gene Census) identified in the targeted data but not in the whole-exome data. In parentheses: p-values of a Fisher's exact test indicating whether the overlap is higher than expected by chance ($p < 0.05$ marked in bold). Cells are empty if no gene was only identified in the targeted data. These results are based on the MSK-IMPACT gene panel.

**Tool**	**BLCA**	**BRCA**	**COAD**	**GBM**	**HNSC**	**KIRC**	**LC**	**LGG**	**OV**	**PRAD**	**SKCM**	**STAD**	**THCA**	**UCEC**
20/20+														
ActiveDriver							0 (1.00)							
dNdScv				**1 (0.02)**			0 (1.00)	0 (1.00)			0 (1.00)	**1 (0.03)**		0 (1.00)
DriverML	0 (1.00)	2 (0.93)	0 (1.00)	1 (0.32)	2 (0.16)	0 (1.00)	**5 (0.01)**	1 (0.34)	0 (1.00)	1 (0.41)	**1 (0.04)**	0 (1.00)	0 (1.00)	0 (1.00)
MutSigCV	0 (1.00)	7 (0.83)	5 (0.84)	2 (0.17)	2 (0.71)	1 (0.35)	3 (0.96)	1 (0.43)	3 (0.35)	1 (0.33)	0 (1.00)	5 (0.20)	0 (1.00)	2 (0.25)
OncodriveCLUSTL	0 (1.00)	1 (0.22)	0 (1.00)	0 (1.00)	0 (1.00)		0 (1.00)			0 (1.00)	0 (1.00)	1 (0.10)		1 (0.10)
OncodriveFML	0 (1.00)			**1 (0.02)**							0 (1.00)			0 (1.00)

Supplementary table 8. Numbers of known driver genes (according to the Cancer Gene Census) identified in the whole-exome data but not in the targeted data. In parentheses: p-values of a Fisher's exact test indicating whether the overlap is higher than expected by chance ($p < 0.05$ marked in bold). Cells are empty if no gene was only identified in the whole-exome data. These results are based on the MSK-IMPACT gene panel.

**Tool**	**BLCA**	**BRCA**	**COAD**	**GBM**	**HNSC**	**KIRC**	**LC**	**LGG**	**OV**	**PRAD**	**SKCM**	**STAD**	**THCA**	**UCEC**
20/20+														
ActiveDriver														
dNdScv	0 (1.00)	0 (1.00)	1 (0.23)	**1 (0.04)**	0 (1.00)	**1 (0.02)**	0 (1.00)	0 (1.00)		**1 (0.01)**		0 (1.00)	0 (1.00)	0 (1.00)
DriverML	1 (0.50)	2 (0.84)	**8 (0.01)**	1 (0.49)	0 (1.00)	0 (1.00)	0 (1.00)	1 (0.75)	2 (0.29)	0 (1.00)	0 (1.00)	2 (0.12)	1 (0.32)	4 (0.23)
MutSigCV	**1 (0.03)**		0 (1.00)	0 (1.00)	0 (1.00)		0 (1.00)	0 (1.00)			**1 (0.04)**	0 (1.00)		**2 (0.01)**
OncodriveCLUSTL	0 (1.00)	0 (1.00)			3 (0.06)		0 (1.00)	2 (0.01)				0 (1.00)		0 (1.00)
OncodriveFML	0 (1.00)				0 (1.00)		0 (1.00)				0 (1.00)			0 (1.00)

Supplementary table 9. Known driver genes (according to the Cancer Gene Census) identified in the whole-exome data but not in the targeted data. Only genes with a nominal p-value > 0.05 in the analysis of the targeted mutation data are shown. The columns p^{TS} and p^{WE} list the p-values obtained in the targeted data and whole-exome data analyses respectively. Mutation frequency refers to the mutation frequency of the gene in the TCGA dataset of the corresponding cancer type.

Tool	Cancer type	Gene	p^{TS}	p^{WE}	Mutation frequency
DriverML	BRCA	HGF	0.19	0.003	0.81%
DriverML	BLCA	NOTCH1	0.11	0.0005	5.15%
DriverML	COAD	MSH6	0.11	0.00001	5.29%
DriverML	COAD	MSH2	0.42	0.0003	6.05%
MutSigCV	SKCM	RAC1	0.26	0.0001	7.36%
MutSigCV	UCEC	FGFR2	0.28	0.00004	18.34%

Supplementary table 10. Known driver genes (according to the Cancer Gene Census) identified in the targeted data but not in the whole-exome data. Only genes with a nominal p-value > 0.05 in the analysis of the whole-exome mutation data are shown. The columns p^{TS} and p^{WE} list the p-values obtained in the targeted data and whole-exome data analyses respectively. Mutation frequency refers to the mutation frequency of the gene in the TCGA dataset of the corresponding cancer type.

Tool	Cancer type	Gene	p^{TS}	p^{WE}	Mutation frequency
MutSigCV	LGG	ACVR1	0.0	0.59	0.20%
MutSigCV	BRCA	PPM1D	1.1e-04	0.17	0.60%
MutSigCV	OV	PPP2R1A	1.1e-04	0.10	1.23%
MutSigCV	BRCA	RAD50	5.2e-05	0.59	1.31%
MutSigCV	LC	AKT1	0.0	0.90	1.32%
MutSigCV	BRCA	MAP3K13	9.4e-06	0.93	1.71%
MutSigCV	OV	ATR	9.8e-05	0.28	1.98%
MutSigCV	HNSC	MTOR	6.9e-05	0.65	1.99%
MutSigCV	BRCA	EP300	0.0	0.35	2.22%
MutSigCV	LC	RAD21	0.0	0.41	2.24%
MutSigCV	BRCA	ARID1B	0.0	0.62	2.62%
MutSigCV	COAD	MAP2K1	7.4e-05	0.32	3.02%
MutSigCV	OV	BRCA2	1.4e-08	0.085	3.70%
MutSigCV	COAD	MAX	0.0	0.22	4.28%
MutSigCV	HNSC	PTPRT	1.1e-03	0.52	4.77%
MutSigCV	STAD	FGFR2	8.0e-04	0.60	5.12%
MutSigCV	STAD	ERBB2	8.4e-13	0.060	6.51%
MutSigCV	COAD	ERBB3	1.7e-12	0.63	6.80%
MutSigCV	STAD	ATR	4.4e-05	0.44	7.67%
MutSigCV	UCEC	MAX	3.6e-06	0.092	8.70%
DriverML	LC	ERBB4	1.4e-04	0.19	11.51%
MutSigCV	STAD	ERBB4	7.1e-04	0.15	13.72%

References

Arnedo-Pac, C., *et al.* OncodriveCLUSTL: a sequence-based clustering method to identify cancer drivers. *Bioinformatics* 2019;35(22):4788-4790.

Han, Y., *et al.* DriverML: a machine learning algorithm for identifying driver genes in cancer sequencing studies. *Nucleic Acids Res* 2019;47(8):e45.

Kircher, M., *et al.* A general framework for estimating the relative pathogenicity of human genetic variants. *Nat Genet* 2014;46(3):310-315.

Lawrence, M.S., *et al.* Mutational heterogeneity in cancer and the search for new cancer-associated genes. *Nature* 2013;499(7457):214-218.

Martincorena, I., *et al.* Universal Patterns of Selection in Cancer and Somatic Tissues. *Cell* 2017;171(5):1029-1041 e1021.

Mularoni, L., *et al.* OncodriveFML: a general framework to identify coding and non-coding regions with cancer driver mutations. *Genome Biol* 2016;17(1):128.

Reimand, J. and Bader, G.D. Systematic analysis of somatic mutations in phosphorylation signaling predicts novel cancer drivers. *Mol Syst Biol* 2013;9:637.

Tokheim, C.J., *et al.* Evaluating the evaluation of cancer driver genes. *Proc Natl Acad Sci U S A* 2016;113(50):14330-14335.

Vogelstein, B., *et al.* Cancer Genome Landscapes. *Science* 2013;339(6127):1546-1558.
